# First record of hybridization between green *Chelonia mydas* and hawksbill *Eretmochelys imbricata* sea turtles in the Southeast Pacific

**DOI:** 10.7717/peerj.1712

**Published:** 2016-02-18

**Authors:** Shaleyla Kelez, Ximena Velez-Zuazo, Aldo S. Pacheco

**Affiliations:** 1 ecOceánica, Lima, Peru; 2 Center for Conservation and Sustainability, Smithsonian Conservation Biology Institute, National Zoological Park, Washington, D.C., United States of America; 3 Instituto de Ciencias Naturales Alexander von Humboldt, Facultad de Ciencias del Mar y de Recursos Biológicos, Universidad de Antofagasta, Antofagasta, Chile

**Keywords:** Hybridization, Cheloniidae, Interspecific breeding, Cytochrome oxidase I, Female Eretmochelys imbricata

## Abstract

Hybridization among sea turtle species has been widely reported in the Atlantic Ocean, but their detection in the Pacific Ocean is limited to just two individual hybrid turtles, in the northern hemisphere. Herein, we report, for the first time in the southeast Pacific, the presence of a sea turtle hybrid between the green turtle *Chelonia mydas* and the hawksbill turtle *Eretmochelys imbricata.* This juvenile sea turtle was captured in northern Peru (4°13′S; 81°10′W) on the 5^th^ of January, 2014. The individual exhibited morphological characteristics of *C. mydas* such as dark green coloration, single pair of pre-frontal scales, four post-orbital scales, and mandibular median ridge, while the presence of two claws in each frontal flipper, and elongated snout resembled the features of *E. imbricata*. In addition to morphological evidence, we confirmed the hybrid status of this animal using genetic analysis of the mitochondrial gene cytochrome oxidase I, which revealed that the hybrid individual resulted from the cross between a female *E. imbricata* and a male *C. mydas*. Our report extends the geographical range of occurrence of hybrid sea turtles in the Pacific Ocean, and is a significant observation of interspecific breeding between one of the world’s most critically endangered populations of sea turtles, the east Pacific *E. imbricata*, and a relatively healthy population, the east Pacific *C. mydas*.

## Introduction

Hybrids of sea turtles are uncommon in nature and they seem to occur only among species of the Cheloniidae family as no hybrids involving leatherback turtle (*Dermochelys coriacea*) have been found (Karl, Bowen & Avise, 1995). As far back as one hundred years ago (Wood, Wood & Critchley, 1983), hybrid individuals were observed to show intermediate morphological traits of species (Wood, Wood & Critchley, 1983). However, due to the high phenotypic plasticity inherent to species morphological traits, biochemical confirmation of the existence of sea turtles hybrids was needed to confirm the occurrence of hybridization. Protein electrophoresis was the earliest technique that proved the existence of hybrids *Chelonia mydas* × *Eretmochelys imbricata* and *Caretta caretta* × *E. imbricata* (Wood, Wood & Critchley, 1983; Conceição et al., 1990), but the use of advanced molecular and genetics assays reveal intercrossing among more species (Karl, Bowen & Avise, 1995). It is thought that sea turtle hybrids occur due to a disproportional abundance of species with similar distribution range and overlapping habitats such as nesting rookeries (Proietti et al., 2014). Also, unequal female:male ratio may also promote mating encounters between individuals of different species (Proietti et al., 2014). In any case, it is unclear whether this is a survival strategy in case of low population numbers or just a natural mechanism of evolution within these marine reptiles. Considering this, reporting the existence of hybrids is important for understanding the prevalence of and the patterns underlying hybridization among sea turtle species.

Although records of sea turtle hybrids exist from several places around the world (Karl, Bowen & Avise, 1995), there are only two reports of hybrids between *C. mydas* × *E. imbricata*, one in the Atlantic (Wood, Wood & Critchley, 1983) and the second one in the Pacific Ocean (Seminoff et al., 2003). In the Atlantic, several of these hybrids were found in a *C. mydas* nest in Suriname, likely the offspring of a female *C. mydas* crossed with a male *E. imbricata*; twenty years later, the Pacific record reported in Seminoff et al. (2003) highlighted that this hybridization is apparently not gender-biased, since theirs was the first observation of hybridization between a male *C. mydas* and a female *E. imbricata.* Herein, we report a new case of such hybridization between *C. mydas* and *E. imbricata* found in northern Peru. The finding adds support to the absence of gender bias between these species and constitutes the first report of a hybrid sea turtle in the Southeast Pacific.

## Methods

### Sampling location

**Figure 1:**
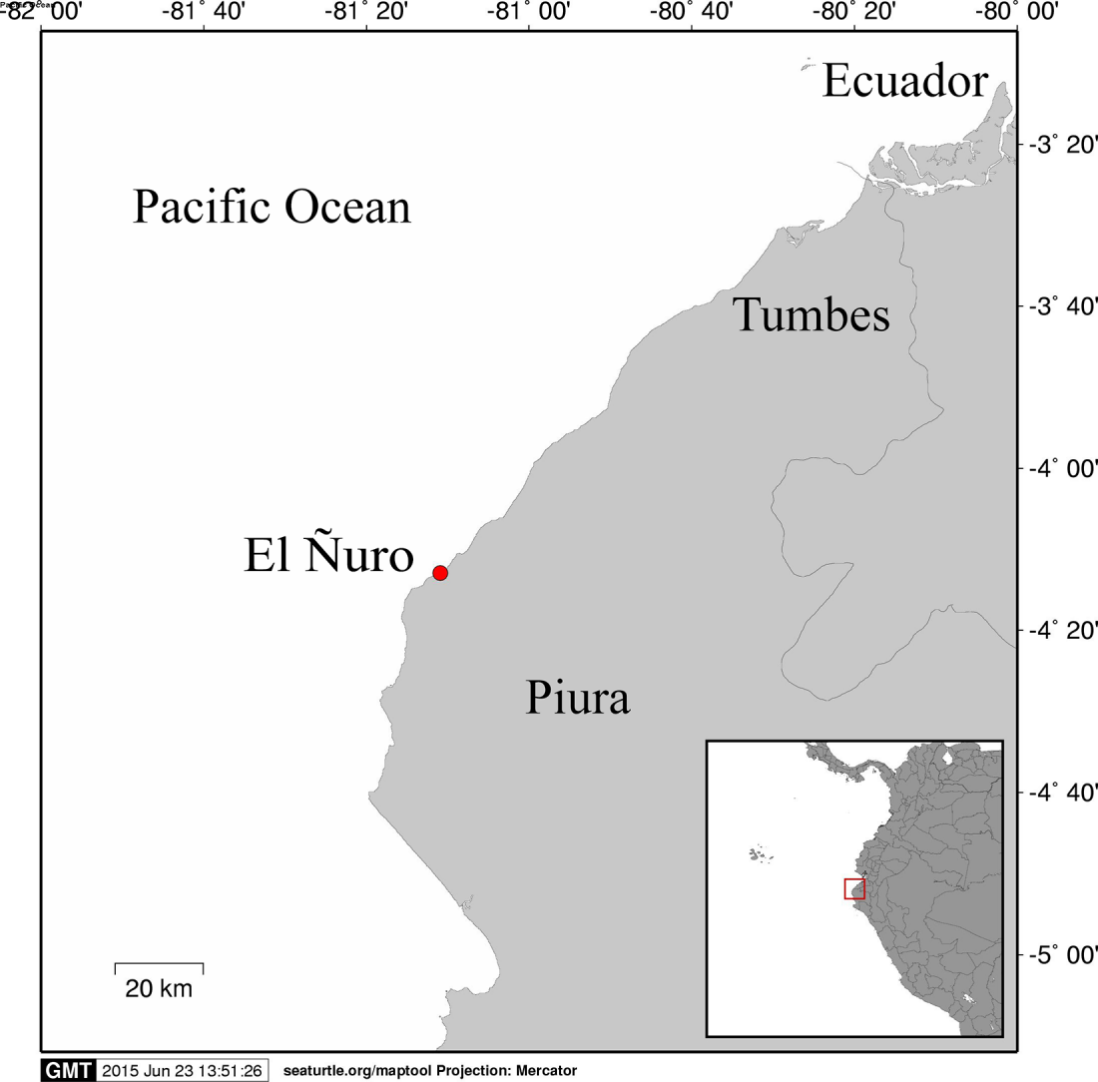
Map of northern Peru showing the location where the hybrid sea turtle was found. SEATURTLE.ORG Maptool. 2002. SEATURTLE.ORG, Inc. http://www.seaturtle.org/maptool/ (July 2015).

The individual was collected during a seasonal green turtle monitoring effort conducted at El Ñuro (4°13′S; 81°10′W, Fig. 1), a large sandy neritic area with rocky reefs in the coast of the department of Piura, northern Peru. The characteristics of the study site and details of the sampling strategy are available in Velez-Zuazo et al. (2014). Permits for the study were granted from the Dirección General Forestal y Fauna Silvestre: RD N°0383-2010-AG-DGFFS-DGEFFS and RD N°0606-2011-AG-DGFFS-DGEFFS.

**Figure 2:**
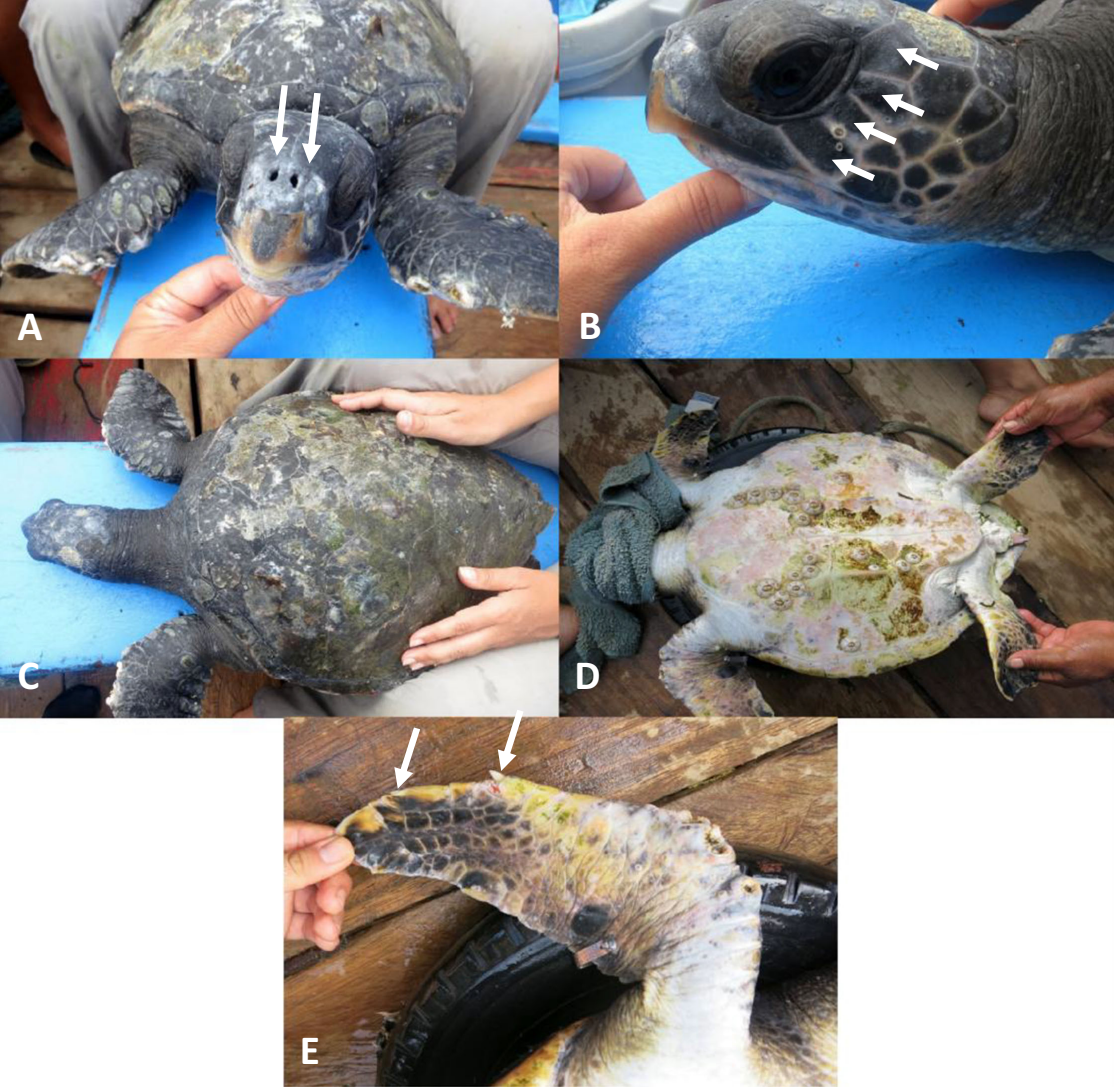
Hybrid individual between *Chelonia mydas* and *Eretmochelys imbricata* captured at El Ñuro, northern Peru. Morphological traits of *Chelonia*; (A) pair of pre-frontal scales, (B) four post-orbitales scales, (C) dark green coloration. Morphological traits of *Eretmochelys*; (C) peeling carapace, (D) pinkish plastron with epibionts, (E) two nails at the flipper.

On the 5^th^ of January, 2014, a small sea turtle was observed surfacing frequently during the first hour of the survey after deploying the entanglement net. At 9:50 AM the individual was caught in the net and was brought on board. During the first visual examination, it was evident that the sea turtle presented morphological characteristics of both *Chelonia mydas* and *Eretmochelys imbricata* (Fig. 2). The following body measurements were taken: notch to tip Curved Carapace Length (CCLn-t) and Curved Carapace Width (CCW), taken with a 100-cm soft measuring tape and 0.1 cm accuracy, notch to tip Straight Carapace Length (SCLn-t) and Straight Carapace Width (SCW) measured with a 100-cm Haglöf tree caliper, and weight, estimated with a 100 kg spring scale. Subsequently, a sample of tissue from the right shoulder area was obtained using a 4 mm-diameter biopsy punch (Acuderm) and stored in 90% ethanol at room temperature. Photographs of all characteristics were taken. Finally, inconel tags with unique identification codes were applied at both front flippers before releasing the individual into the water. The turtle was recaptured at 08:55 am on the following day, and released again after taken additional photographs.

### Molecular analysis

To identify the maternal lineage of our individual and the likely origin of the mother we conducted a phylogenetic reconstruction analyzing the nucleotide variation in the gene cytochrome oxidase I (cox1) from the mitochondrial DNA (mtDNA). Whole genomic DNA was isolated using a Qiagen DNeasy blood and tissue kit according to manufacturer’s instructions and eluted in 50 μl of buffer AE (Qiagen, Valencia, CA, USA). Approximately 679 base-pairs of cox1 were targeted and amplified through Polymerase Chain Reaction (PCR), using specific primers (M13-tailed cocktail primers Fish-F1t1and Fish-R1t1, Ivanova et al., 2007). PCR conditions for an 8 μl amplification product were as follow: 1 μl of genomic DNA at a concentration of ∼20 ng/μl, 5 μl of Taq Master Mix (Qiagen, Valencia, CA, USA), 0.5 μl of each 10 uM primer cocktail and ultrapure water. Cycling conditions were an initial denaturing step (94 °C for 2 min) followed by 35 cycles of 30 s at 94 °C, 40 s at 52 °C, and 1 min at 72 °C, and a final extension of 10 min at 72 °C. The amplification product was purified using a phosphatase and exonuclease and sequencing of both strands was conducted using an automated station ABI 3130xl sequencer (Applied Biosystems, Foster City, CA, USA). Both forward and reverse sequences were edited using Sequencher 4.8 (Gene Codes) and aligned with sea turtle species baseline sequences downloaded from the Barcode of Life Project (BOLD, www.boldsystems.org).

To conduct the phylogenetic reconstruction we used two approaches. First, we used BOLD Identification System to compare our sequence to the species level barcode records. Identification using this method relies in a Neighbor-Joining analysis. Second, we downloaded barcode sequences for all extant species of sea turtle and from different regions of the world (See Supplementary Information). All sequences, including the sequence from our specimen, were aligned using ClustalW, with default parameters, as implemented in Mega 5.05 (Tamura et al., 2011). A Maximum Likelihood (ML) algorithm implemented in RAxML (Stamatakis, 2014) using the GTR model of nucleotide substitution with GAMMA correction and a rapid Bootstrap analysis using the auto MRE bootstopping criterion was used. We set the leatherback sea turtle (*Dermochelys coriacea*) sequence as outgroup for the phylogenetic inference. Figtree v1.4.0 (http://tree.bio.ed.ac.uk/software/figtree/) to draw the consensus tree and for including values of branch support for all analyses.

## Results

### Morphological characteristics

The hybrid sea turtle measured 47.3 cm CCLn-t, 42.3 cm CCW, 44.6 cm SCLn-t, 34.1 cm SCW, and weighted 11 kg, and it was categorized as a juvenile since *Chelonia mydas* and *Eretmochelys imbricata* reach sexual maturity at >85 and 78.8 cm CCL respectively (Liles et al., 2011; Zárate et al., 2013). Most of the external morphological characteristics resemble those of *C. mydas* such as dark green coloration of the carapace and skin, non-serrated marginal carapace, pair of pre-frontal scales, four post-orbital scales (Fig. 2 and Table 1), and the presence of a median ridge in the lower jaw. Visible *E. imbricata* characteristics were a pair of claws on each front flipper, long beak, non-imbricated peeling scutes, plastron with pink coloration, and with high presence of epibionts. High density of epibionts on the plastron were previously observed in *E. imbricata* captured at El Ñuro, but such densities of barnacles are uncommon on *C. mydas* at this site (Kelez et al., 2010–2014, personal observations).

**Table 1:** Comparison of morphological traits among hawksbill, green and hybrid turtles including the individual found at El Ñuro.

	Hawksbill	Hybrids			Green
Characteristics		Suriname^1^	Mexico^2^	Peru^3^	
Imbricated scutes	Yes	Yes	Yes	No	No
Elongated snout	Yes	Yes	Yes	Yes	No
Second claw	Yes	Yes	No	Yes	No
Marginal scute serration	Yes	Yes	Yes	No	No
Pre-frontal scales	Four	Variable	Two	Two	Two
Post-orbital scales	Three	Not reported	Three	Four	Four
Mandibular middle ridge	No	Yes	Yes	Yes	Yes

**Notes:**

1 Wood, Wood & Critchley (1983).

2 Seminoff et al. (2003).

3 This study.

### Maternal lineage identification and origin

**Figure 3:**
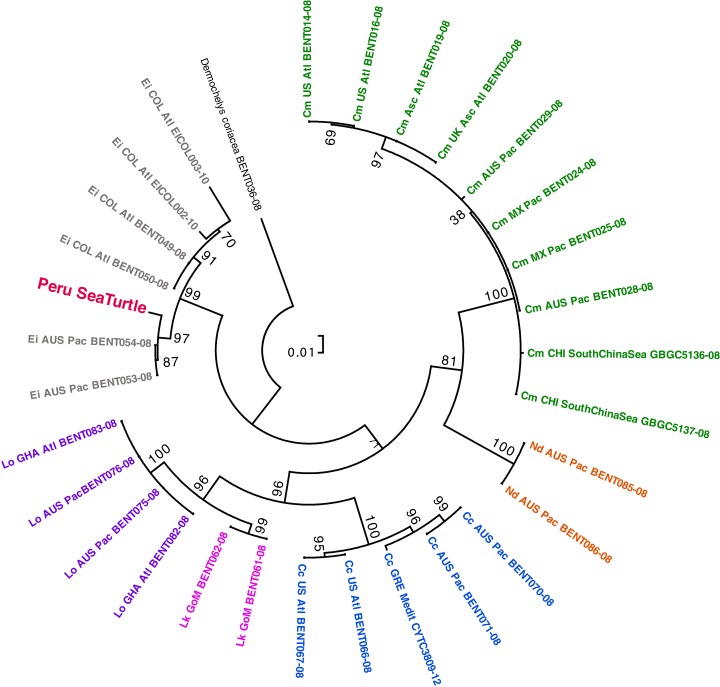
Phylogenetic placement of the hybrid specimen as suggested after a Maximum Likelihood analysis. Species were color-labeled by genus and are *Chelonia mydas* (green), *Natator depressus* (orange), *Caretta caretta* (blue), *Lepidochelys kempii* (fucsia), *Lepidochelys olivacea* (purple), and *Eretmochelys imbricata* (gray). The specimen under study is indicated with a dark pink label.

We obtained a 717bp sequence of mtDNA cox1 that we used for phylogenetic tree reconstruction (GenBank accession number KU254594). Both, the NJ phylogenetic used by BOLD and the ML phylogenetic tree we reconstructed placed our specimen within the *Eretmochelys imbricata* clade. The results provided by BOLD species identification, indicated that the sequence of our individual matched to *E. imbricata* with a probability interval of 93.22–99.57% at the species level, and 100% at the genus level. Similarly, the ML phylogenetic reconstruction placed our specimen within the *E. imbricata* clade (99% node support for ML consensus tree), specifically within the clade composed of individuals from populations from the Pacific Ocean basin (97% node support for ML consensus tree) (Fig. 3).

## Discussion

To the best of our knowledge, this is the first time a hybrid sea turtle has been reported in the southeast Pacific. Furthermore, it represents the second case of a crossing between a female *Eretmochelys imbricata* and a male *Chelonia mydas* in the entire Pacific Ocean, and only the third case in all oceans. Morphologically, the appearance of the hybrid individual mostly resembled that of *C. mydas*; however, the comparison with the rest of *E. imbricata* and *C. mydas* hybrids suggest that only the mandibular middle ridge from *C. mydas* and the elongated snout from *E. imbricata* is present in hybrids, while other morphological traits are somehow variable (Table 1). Because the individual generally resembled a *C. mydas*, it may be possible that the existence of more hybrids is going unnoticed without careful morphological evaluation. In addition, we suggest that whenever possible, skin samples for molecular analyses should be taken from individuals with indistinct morphological characteristics.

Difference in abundance between species has been proposed as a condition that may promote hybridization. Nesting numbers in *C. mydas* rookeries had been recovering over the last 30 years in the eastern Pacific, and are significantly higher when compared to the *E. imbricata* eastern Pacific nesting regions (Seminoff et al., 2012). In fact, due to its small size and the numerous threats it faces, the eastern Pacific *E. imbricata* is one of the eleven most endangered sea turtle populations in the world (Wallace et al., 2011). This contrast between populations is evidenced in foraging aggregations of sea turtles in northern Peru. From 2010 to 2014, during seasonal (n = 11) in-water surveys at El Ñuro, a total of 186 different individual *C. mydas* were captured but only two *E. imbricata* were observed (Velez-Zuazo et al., 2014). Also, for both species, foraging and nesting habitat overlap occurs to some degree in the eastern Pacific. Both species are distributed throughout the same coastal line and can be found in algal seabed, rocky reefs and especially in mangrove habitats, as recently reported in Gaos et al. (2012). However, there are no rookeries in Peru and also adult *E. imbricata* are uncommon in Peruvian waters (Hays-Brown & Brown, 1982; Kelez, Velez-Zuazo & Manrique, 2003; Alfaro-Shigueto et al., 2010; Rosales, Vera & Llanos, 2010; Quiñones et al., 2011); all but one of the *E. imbricata* reported in Peru have been juveniles (Forsberg, 2008). Therefore, it is likely that mating of this hybrid’s progenitors occurred in waters to the north of Peru where *E. imbricata* rookeries are located (i.e., Ecuador, Costa Rica, Nicaragua, El Salvador, Mexico). Moreover, given that both *E. imbricata* × *C. mydas* hybrids reported so far in the eastern Pacific resulted from a crossing between a male *C. mydas* and a female *E. imbricata*, it is possible that male *E. imbricata* are available in insufficient numbers to breed during reproductive seasons due to the reduced population size of the eastern Pacific *E. imbricata*. For these reasons, we hypothesize that, the current population status and habitat overlap may be the reason for the existence of hybrids of these two species in the southeast Pacific Ocean.

Taxonomically, *C. mydas* and *E. imbricata* belong to the Chelonini and Carettini tribes, respectively, and were phylogenetically separated more than 50 millions of years ago (Karl, Bowen & Avise, 1995). Therefore, this hybridization case clearly shows interbreeding between ancient sea turtles linages, an event reported for other populations. For example, at the Bahia rookery (Brazil, Southwest Atlantic) 42% of sampled females identified as hawksbills were actually hybrids between *Caretta caretta* and *E. imbricata*; these hybrids are fertile and reproductively successful (Lara-Ruiz et al., 2006). This brings over the table a previous discussion about what is the most appropriate scheme for taxonomic classification of species (Avise & Johns, 1999). Although we were unable to ascertain the generation and fertility of the hybrid captured in northern Peru, we cannot overrule that reproduction between hybrids could be occurring. We recommend that additional research be conducted to further identify hybrid individuals in the eastern Pacific, as well as estimate their abundance, fertility and the ecological role they are playing in marine ecosystems.

## Supplemental Information

10.7717/peerj.1712/supp-1 Supplemental Information 1 Sequences used for phylogenetic analyses. Supplementary Data. Sequences used for phylogenetic analyses. All sequences were downloaded from the Barcode of Life Database System, except for the sequence obtained in this study.

## Additional Information and Declarations

### Field Study Permissions

The following information was supplied relating to field study approvals (i.e., approving body and any reference numbers): We were granted permits from the Direccion Gerenal Forestal y Fauna Silvestre: RD N°0383-2010-AG-DGFFS-DGEFFS and RD N°0606-2011-AG-DGFFS-DGEFFS.

### DNA Deposition

The following information was supplied regarding the deposition of DNA sequences: GenBank Accession Number KU254594.
